# Development of a posterior sagittal anorectal surgical teaching model

**DOI:** 10.1186/s12887-021-02514-5

**Published:** 2021-01-27

**Authors:** J. A. van Ling, G. M. J. Bökkerink, I. de Blaauw, S. M. B. I. Botden

**Affiliations:** 1 Department of Pediatric Surgery, Radboudumc – Amalia Children’s Hospital, Route 618, Nijmegen, 6500 HB the Netherlands; 2 Department of Pediatric Surgery, Princess Máxima Center for Paediatric Oncology, Utrecht, the Netherlands

**Keywords:** Anorectal malformations, Posterior sagittal anorectoplasty, Surgical training, Inanimate model

## Abstract

**Background:**

An Anorectal Malformation (ARM) is a rare congenital malformation, which requires proper correction to ensure the best long-term prognosis. These procedures are relatively infrequent and complex, in which a structured approach is important. Therefore, training on an affordable model could be beneficial.

**Methods:**

A low-cost ARM model was developed. The base was reusable and the perineal body disposable. Both expert pediatric surgeons (Experts) and residents/fellows (Target group) were recruited for this study. After testing the model, they completed a questionnaire regarding the realism and didactic value of the model, using a 5-point Likert scale.

**Results:**

Forty-four participants were recruited (Target group *n* = 20, Experts *n* = 24). The model has high mean scores of 3.8–4.4 for the total group and even higher on several aspects by the Target group. The experts regarded the haptics and manipulation of the fistula less realistic than the Target group (3.7 versus 4.3, *p* = 0.021 and 4.2 versus 4.6, *p* = 0.047). It was considered to be a very good training tool (mean 4.3), without significant differences between the groups.

**Conclusions:**

These results show general consensus that this model is a potent training tool for the component steps of the repair of an ARM with recto-perineal fistula by sagittal approach.

## Background

An Anorectal Malformation (ARM) is a rare congenital malformation characterized by abnormal or absent development of the anus and distal rectum. There is a wide spectrum of clinical presentations, ranging from ARM with rectoperineal- or vestibular fistula to ARM with bladderneck fistula or cloaca [1, 2]. The Posterior Sagittal Anorectoplasty (PSARP) or Anterior Sagittal Anorectoplasty (ASARP) are the most common procedures in the treatment for ARM with rectoperineal or vestibular fistulas and consist of several systematized component steps for reconstruction of the recto-perineal complex [1, 3, 4]. From the introduction in the early 1980s, these procedures have become popular for the reconstruction of an ARM and has improved patient outcomes ever since [5]. Important with this relatively infrequent and complex procedure is a structured approach to ensure a positive clinical outcome [5]. Suboptimal performance of the procedure can result in unfavorable outcomes, such as wound dehiscence, infections and even non-proper localization of the neo-anus, causing incontinence and severe constipation.

To acquire and retain these specific procedural skills, sufficient training is needed for pediatric surgical residents, fellows and young pediatric surgeons. This training is traditionally based on the apprenticeships model, which allows trainees to practice on patients under supervision [6]. Another option is a one day or half-day hands-on course to train this procedure on a (live) animal model, which is expensive, recourses are limited and not readily available. Moreover, creating anatomical variation remains a challenge and the use of live animals is an ethical issue [7, 8]. Also, practicing one procedure is probably not enough for a proper procedural skills training. Having an artificial training model, especially for relatively rarely performed procedures, is beneficial for training the component steps and ensures a more routine approach in the clinic setting. Simulation-based training has become increasingly popular over the past decade and allows trainees to train for surgical skills outside the clinical setting [9, 10]. However, no data was found on simulation-based training for an ARM and as far as we know, no models are currently available.

In this study we developed a low-budget, transportable model to systematically train the procedural steps of the repair of an ARM with perineal fistula, by sagittal approach, outside the clinical setting. The aim of this study was to validate the model for the training of pediatric surgery trainees.

## Methods

### Development of the ARM model

**Fig. 1:**
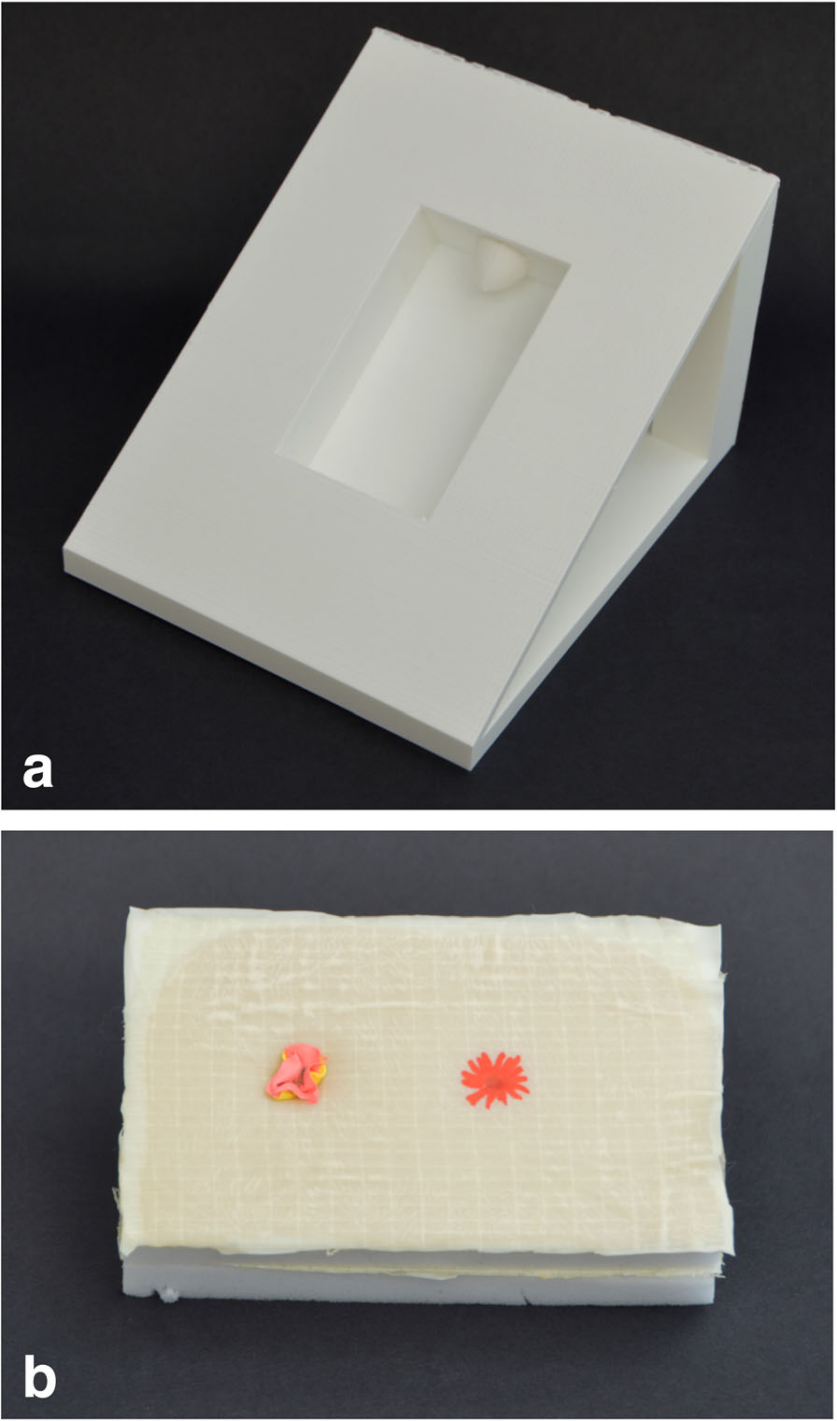
**a** Case **b** Sponge representing the perineal body

The model was made to simulate the perineum of a patient during the PSARP/ASARP procedure, lying in prone (jackknife) position. The base of the model is a triangular shaped casing, with a box centrally, including the coccygeal bone cranially, which was reusable (Fig. 1a).

The disposable perineal body consisted of a 5x10x2cm melanine sponge which is divided over the length into two equally sized parts. Different materials were applied on top and between the sponges to create different layers and thus to simulate the perineal anatomy as much as possible (Fig. 1b):

- Layer 1: Skin – surgical non latex gloves (©Sempermed) and Duoderm (©Convatec)
- Layer 2: Subcutaneous fat – sponge no. 1
- Layer 3: Fascial layer – surgical non latex gloves (©Sempermed)
- Layer 4: Rectal fistula – double layered balloon (glued to each other and the sponge)
- Layer 5: Perirectal fat – sponge no. 2

The layers were glued together with specialized acrylic glue. When finished, the end product (disposable sponge) was inserted in the box of the reusable base (Fig. 1a and b).

During the development of the model, multiple requirements were set to which the model needed to comply. First, the model needed to be affordable. To establish this, the perineal body was made of multiple surgical and non-surgical disposables. Second, the production of the model needed to be fairly simple and reproducible. This was accomplished by creating a reusable 3D-printed case and a disposable perineal body which was easy to produce with some non-complex but very precise steps. However, this still took approximately 30 min per sponge to create. Third, the model needed to be a valid training tool for the component steps of a PSARP/ ASARP.

**Fig. 2:**
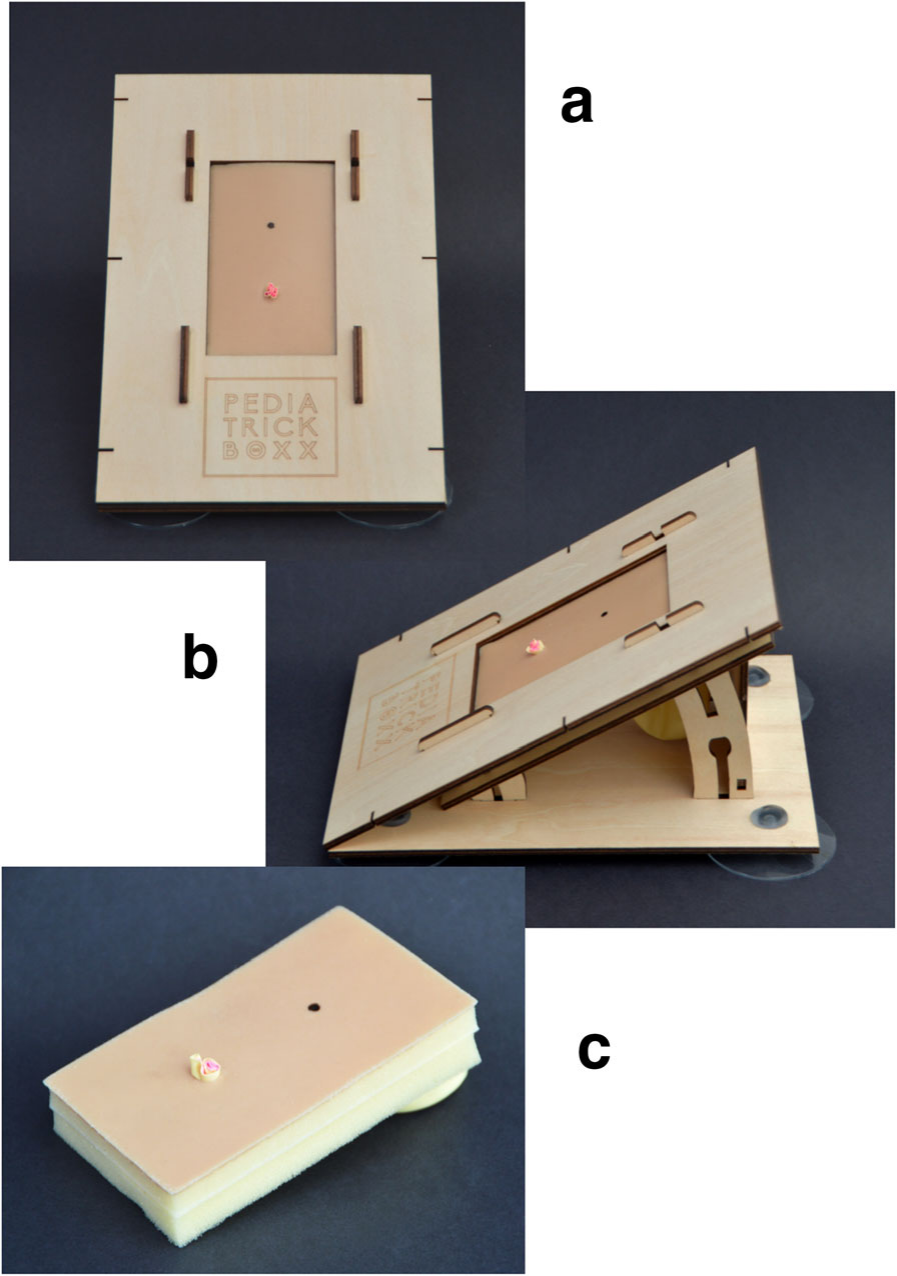
**a** and **b** Improved version of the case. **c** Improved version of the sponge representing the perineal body

After the evaluation process, a second version of the model was created to make it cheaper and easier to produce, without losing the features that were validated. This led to the end-product of the perineal body sponge that met up with the previously stated requirements (Fig. 2c). The case of the model developed during this study, was a 3D printed plastic version in the validation process. However, this would be time a relatively expensive and time-consuming production method. Therefore, to make the product universally available, a second and improved version of the case was developed, after this validation study (Fig. 2a and b). The production of this case will be by laser cutting of wooden plates, which then can be self-assembled to form the case. The purpose of this improved version is to create a ‘take-home version’, in which the case is more suitable to send by post and use in the home or office setting. This way pediatric surgical trainees around the world can train outside the clinical setting, without the restrictions of the need of a skills center.

### Steps of PSARP

The PSARP, used as training procedure in this study, was divided into component steps and as many steps as possible were incorporated to train on the model [4]. The following five steps can be trained:

- Step 1: Placing sutures around fistula
- Step 2: Sagittal opening in midline
- Step 3: Dissection fistula/ rectum
- Step 4: Reconstruction of sphincter complex
- Step 5: Anoplasty

**Fig. 3:**
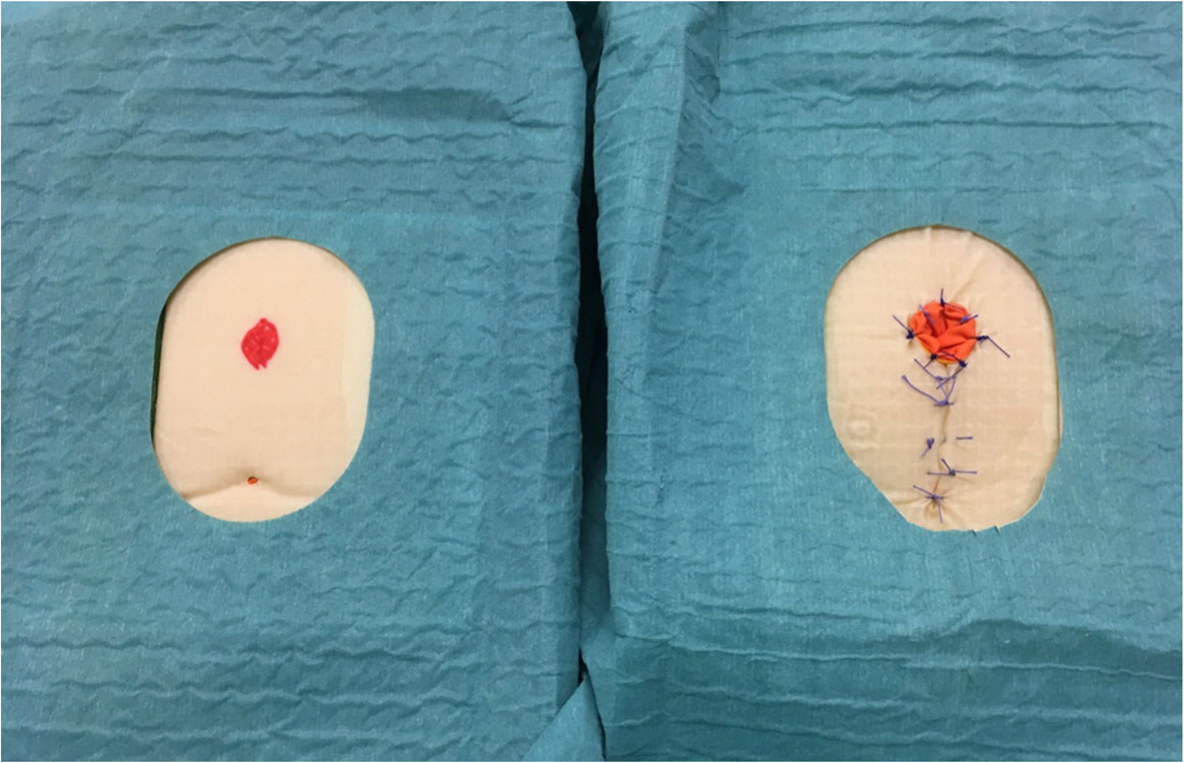
The model with the perineal sponge as used during the training. On the right is after the training of the PSARP procedure

**Fig. 4:**
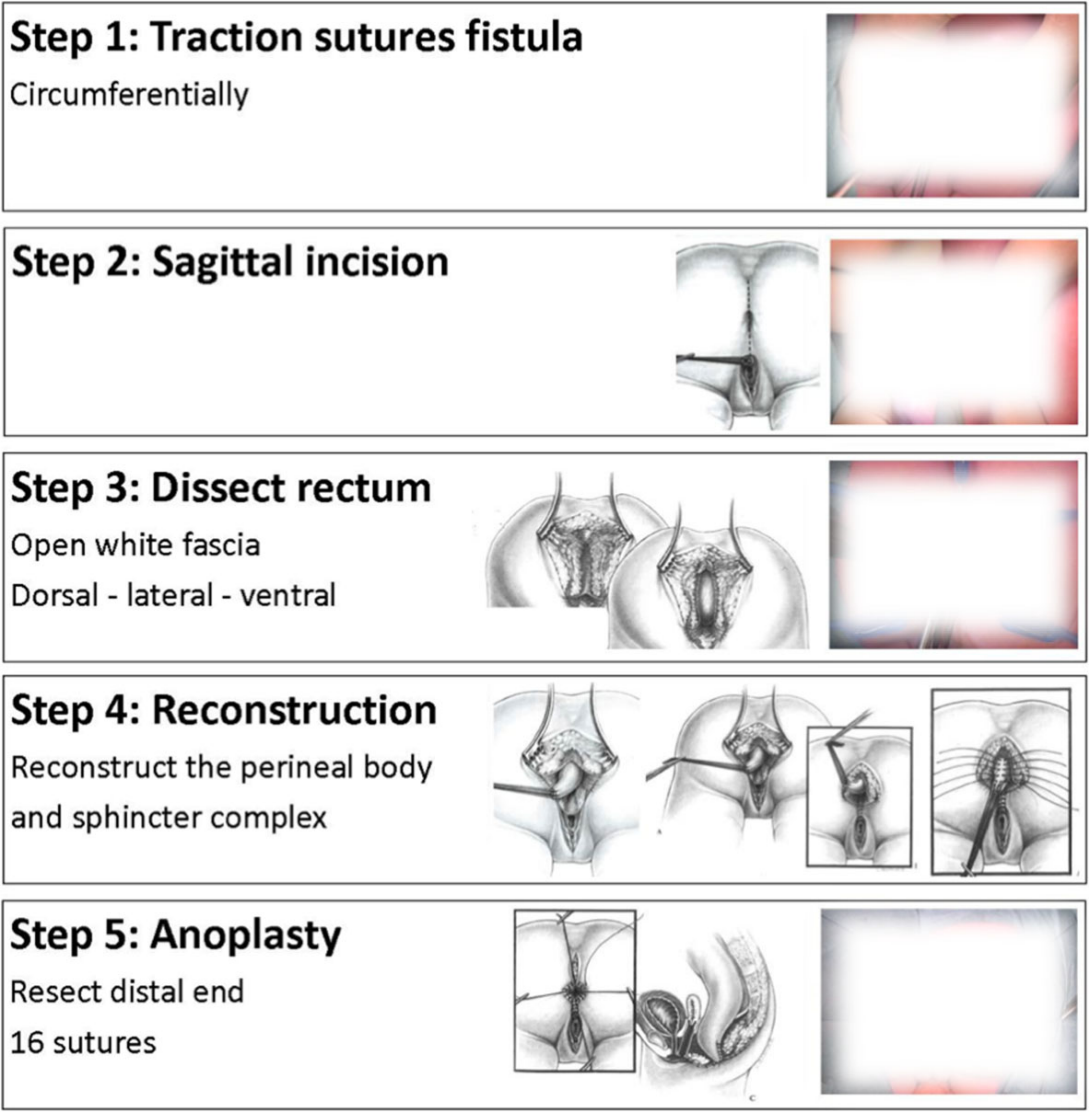
Poster with component steps of a proper Posterior Sagittal Anorectoplasty (PSARP). The drawings in this poster are reprinted from Pena et al. [11]. The photos are blurred for privacy reasons

**Fig. 5:**
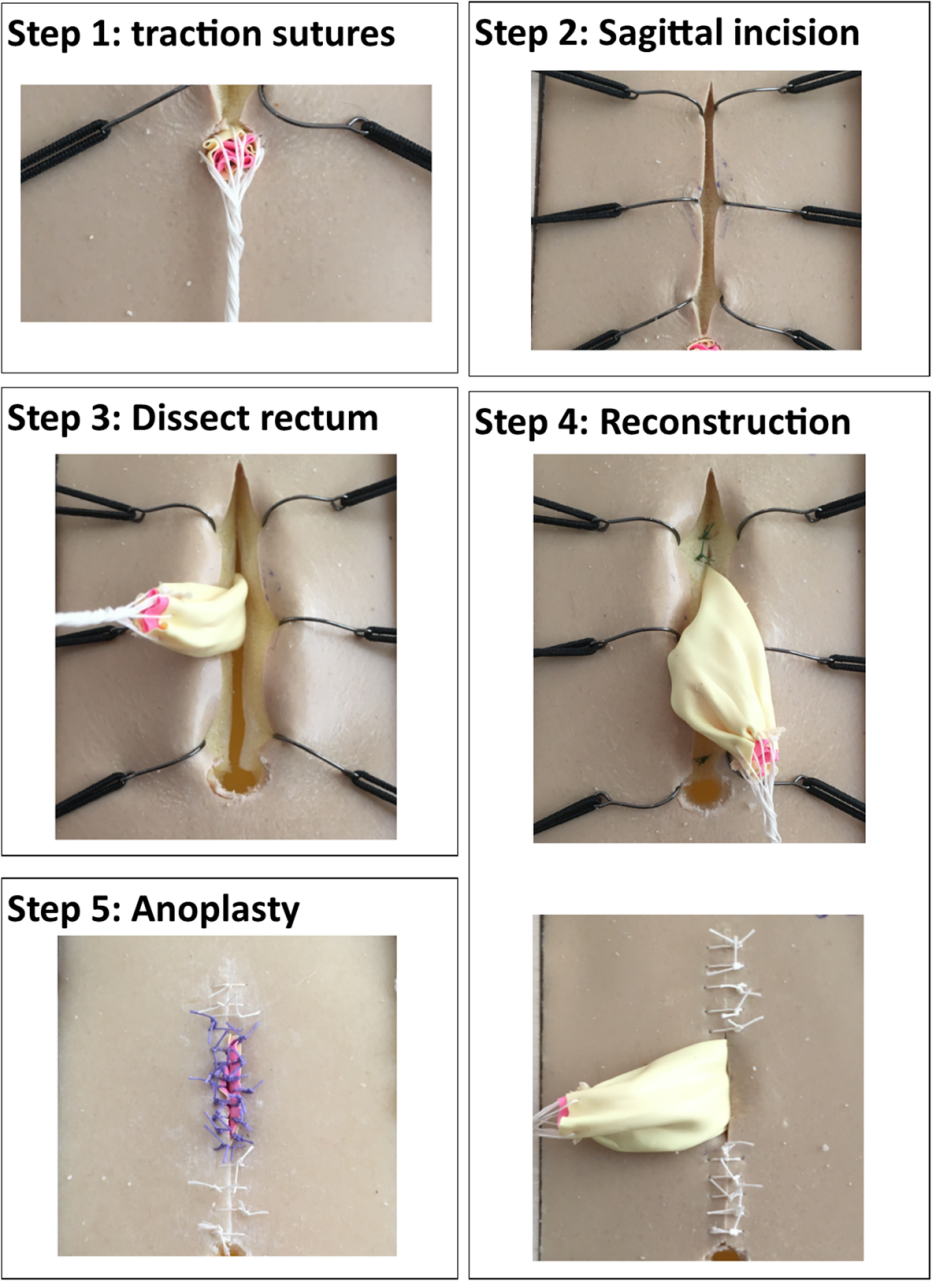
The steps of the procedure performed on the improved version of the ARM model

The focus of the model is the structured approach, because realistic dissection is difficult to simulate, especially in a low-cost model (Fig. 3). Following a poster was developed, of the trainable steps, to guide the trainees during the training on the model (Fig. 4). Figure 5 shows the steps performed on the model.

### Participants

The participants were recruited during the 11th Pediatric Colorectal Congress, Nijmegen the Netherlands, 5-7th December 2018. The subjects were divided into two groups based on their self-reported colorectal experience: ‘target group’ with clinical pediatric surgery experience, but had performed less than twenty PSARP/ ASARP procedures, consisting of pediatric surgery residents, fellows and young pediatric surgeons. The ‘experts’ had performed at least twenty PSARP/ ASARP procedures or had performed between five and ten PSARP/ ASARP procedures and assisted on at least ten PSARP/ ASARP procedures, therefore consisting of senior pediatric colorectal surgeons.

In the validation of new training model it is not only important to know what the experts think of it, but also what the target groups opinion on the training tool is, because they are the ones that have to train on it.

### Questionnaire

The questionnaire was adapted from a previously used questionnaire in other validation studies [7, 12, 13]. It consisted of two parts, with the first part containing the consent, demographics and the participant’s current pediatric surgical experience. The second part of the questionnaire consisted of questions on the realism (regarding realistic response and tissue behaviour) and didactic value (value to train pediatric surgeons) of this model. Each separate task was rated on the five-point-Likert scale. With ‘1’ resulting in strong disagreement, ‘3’ being the neutral opinion and ‘5’ resembling a strong agreement.

### Protocol

After completing the first part of the questionnaire, all participants completed a written informed consent to participate in this study and no ethical approval was needed for this study, which complies with the national regulations at the time of the study.

The participants were asked to train the steps of the procedures on the model, based on the instructions on the poster (Fig. 4). At least one expert per three training models was available for instructions and supervision. If available, the participants were assisted by their peers, because assistance is necessary in the clinical setting as well. All participants were asked for their opinion on the questionnaire, after the completion of the first part on their demographics and previous clinical experience.

### Statistical analysis

The primary outcome measure in this study was the potential of the ARM-model to be a potent training tool for the component steps of a PSARP/ ASARP. Secondary outcome measures included the visual appearance and realism of the model.

The analysis was performed using Statistical Package for Social Sciences (SPSS) version 25. The results of the questionnaires were entered in the database anonymously and compared between the expertise groups, using a Mann-Whitney U test, with a *p*-value of < 0.05 considered a statistically significant difference. The mean ratings were also tested against a neutral opinion (3 on 5-point Likert scale) using a one sample t-test, in which a mean of 3.5 was considered a significantly better result than neutral, indicating an adequate training tool and a mean of > 4.0 was considered a good training tool to use in future training of pediatric surgeons.

## Results

### Participants demographics and clinical experience

During the 11th Pediatric Colorectal Congress, a total of 44 participants were recruited, all with pediatric surgical experience. As shown in Table 1, the target group consisted of 20 participants and the expert group of 24. There was an equal distribution of men and women between the groups (*p* = 0.76). The mean age was higher in the expert group compared to the target group (35 versus 47 years, *p* = < 0.001). The participants of this study consisted of mostly pediatric surgeons (*n* = 29) and pediatric surgery fellows (*n* = 9), but also one pediatric urologist, four pediatric surgical residents and one pediatric surgical intern completed the questionnaires. Three participants, all included in the target group, did not complete their specific colorectal experience, however, two were junior pediatric surgeons and one a pediatric surgery fellow, therefore a minimum level of clinical experience in pediatric surgery was assumed. All participants (both experts and target group) completed the questions on the questionnaire regarding the realism (face validity), training of the steps and the model as a training tool (content validity).

**Table 1:** Demographics both study groups

	Target group (*n* = 20)	Expert group (*n* = 24)
Age, mean (SD)	34.9^a^ (5.7)	47.4^b^ (10.8)
Gender, n (%)		
Female	10^c^ (44)	10^a^ (50)
Male	8^c^ (56)	10^a^ (50)
Profession, n (%)		
Medical intern	1 (5)	0 (0)
Surgical resident	4 (20)	0 (0)
Fellow pediatric surgery	8 (40)	1 (4)
Pediatric surgeon	7 (35)	22 (91)
Pediatric urologist	0 (0)	1 (4)
Colorectal experience (primary), n (%)		
None	8^b^ (40)	0 (0)
< 5 procedures	8^b^ (40)	0 (0)
5–20 procedures	1^b^ (5)	9 (38)
21–50 procedures	0^b^ (0)	5 (21)
> 50 procedures	0^b^ (0)	10 (42)
Colorectal experience (assisted), n (%)		
None	2^b^ (10)	0 (0)
< 5 procedures	2^b^ (10)	2 (8)
5–20 procedures	11^b^ (55)	6 (25)
21–50 procedures	2^b^ (10)	6 (25)
> 50 procedures	0^b^ (0)	10 (41)

Missing values: ^a^4, ^b^3, ^c^2

### Realism of the model (face validity)

**Fig. 6:**
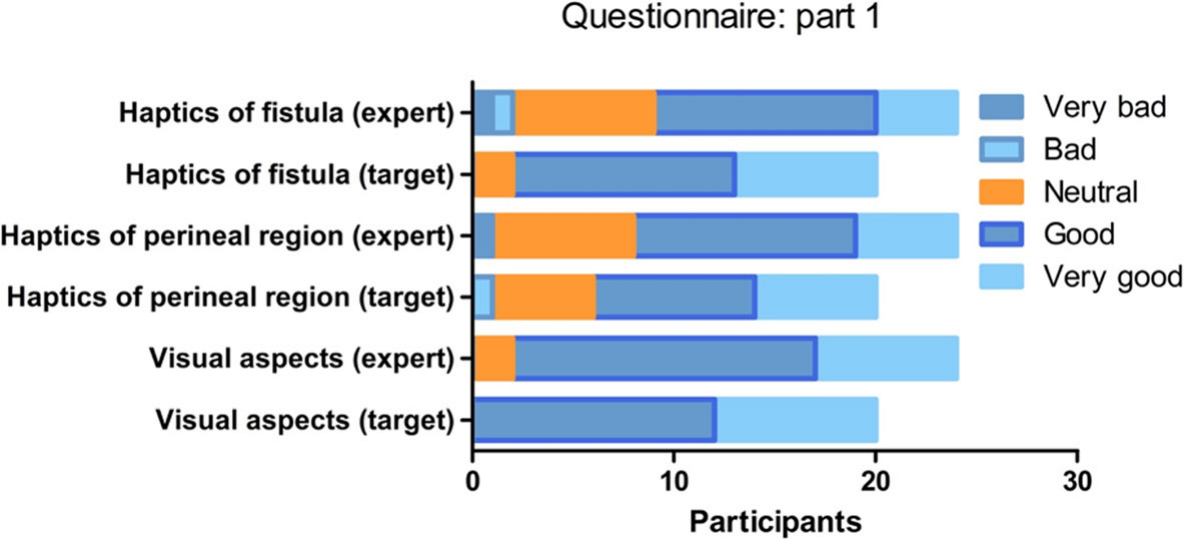
Part 1 of the questionnaire

The responses on the first part of the questionnaire, on the visual appearance and haptics of the model, are visualized in Fig. 6. The overall mean score of 4.3 (SD 0.55) on the visual aspects of the model, without significant differences between the target and experienced group, indicates a satisfying visual presentation of the model. The haptics of the fistula scored significantly lower scored by the experts, who regarded it as less realistic than the target group (3.7 versus 4.3, *p* = 0.02).

### Training of the steps

The responses regarding the possibility to train the component steps of the PSARP on this model are presented Table 2. The mean total scores were over 4.0 for step 1 (placing the sutures around the fistula), step 2 (sagittal opening in midline), step 4 (dissection fistula/rectum) and step 5 (anoplasty), showing an overall positive result on almost all tasks. Only step 3 (dissection of the fistula and rectum) scored lower (mean 3.8, SD = 1.10), although this was still significantly better than a neutral score (*p* < 0.001). There were significant differences in scores between the groups for step 1 (*p* = < 0.04), step 2 (*p* = 0.01) and step 5 (*p* = 0.04), where the experts granted lower scores than the target group.

**Table 2:** Results of questionnaire

Opinions on PSARP-model *Mean (SD)* *Median (range)*	Total group (*n* = 44)	Target group (*n* = 20)	Expert group (*n* = 24)	*P*-value
Visual aspects	4.3 (0.55)	4.4 (0.50)	4.2 (0.59)	0.29
	4 (3–5)	4 (4–5)	4 (3–5)	
Haptics of perineal region	3.9 (0.91)	4.0 (0.89)	3.8 (0.93)	0.61
	4 (1–5)	4 (2–5)	4 (1–5)	
Haptics of fistula	3.9 (0.87)	4.3 (0.64)	3.7 (0.96)	**0.03**
	4 (1–5)	4 (2–5)	4 (1–5)	
Step 1: placing sutures around fistula	4.3 (0.65)	4.6 (0.61)	4.2 (0.64)	**0.04**
	4 (3–5)	5 (3–5)	4 (3–5)	
Step 2: sagittal opening in midline	4.3 (0.77)	4.6 (0.60)	4.0 (0.81)	**0.01**
	4 (2–5)	5 (3–5)	4 (2–5)	
Step 3: dissection fistula/rectum	3.8 (1.10)	3.9 (1.25)	3.8 (0.98)	0.42
	4 (1–5)	4 (1–5)	4 (1–5)	
Step 4: building the sphincter complex	4.1 (0.87)	4.0 (1.02)	4.1 (0.73)	0.98
	4 (2–5)	4 (2–5)	4 (2–5)	
Step 5: anoplasty	4.4 (0.69)	4.6 (0.60)	4.2 (0.72)	**0.04**
	4 (3–5)	5 (3–5)	4 (3–5)	
Training tool for component steps of PSARP	4.3 (0.64)	4.5 (0.61)	4.2 (0.65)	0.09
	4 (3–5)	5 (3–5)	4 (3–5)	

Data in this table represent respectively the mean value (standard deviation) and the median (range). Statistical differences were calculated with the Mann-Whitney U test. A *p*-value < 0.05 was considered significant

### Training tool (content validity)

The last question assessed whether the ARM-model can be considered a good training tool for the component steps of a PSARP. With a mean score of 4.3 (SD = 0.64) in the total group, this was significantly better than 4.0 (*p* = 0.002), without a significant difference between the expertise groups (Table 2).

### Affordability and availability

The ARM-model will be sold in a package. The total price of the package with five reusable sponges, a wooden case and access to the application with video guidance will be approximately €70–100,- and is non-profit (www.PediaTrickBoxx.com). The aim is to have the possibility to purchase the necessary surgical instruments and equipment (low budget) together with the model as well, to ensure that everyone will be able to train in their own setting.

## Discussion

This study is the first to validate an inanimate pediatric surgical training model for the PSARP/ ASARP procedure. Moreover, to our knowledge, there are currently no other ARM training models available on the market. The results of this study indicate that the ARM model developed in this study can be considered a good training tool for the component steps of the PSARP/ ASARP, with a mean score of 4.3. There was a general consensus between the expertise levels that this ARM model is a good training tool for all trained PSARP steps in this study. This makes this model a completely new asset in pediatric surgical training.

It is visible in the results that the target group in fact scored the model better that the expert group, indicating that the target group has less knowledge on the actual haptics of an anorectal malformation. For the face and content validity it is not necessary to have the opinion of the target group. But to evaluate whether it will be used in the home training setting, it is crucial in our opinion, to make sure it is attractive and realistic enough to the less trained pediatric surgical trainee as well. Although the experts rated the model significantly lower than the target group, this was still means of 4.0–4.2 for the training on the model and 3.7–3.8 for the haptics, which are fairly realistic and better than expected for a low budget, reusable training model.

Comparing of the ARM model with traditional animal tissue models, which are nowadays mostly used in wet labs to practice PSARP/ ASARP procedures, there are several advantages as well as disadvantages in the use of an artificial model. Animal models have the advantage of approaching realistic haptic feedback and practicing procedures in a live animal provides almost the same situation as with live surgery [7]. However, the creation of anatomical variances in animal models remains complicated and the resources are limited. Not to mention the fact that the use of live animals is an ethical issue and that the aim is to limit the number of animals used in scientific practice. Artificial models, on the other hand, have the advantage of unlimited conservation, the materials are reusable and various anatomical variances can be created. In addition, artificial models are readily available and can easily be distributed to trainees. Both methods have their own advantages, but artificial models are a potent additional tool in the training of young surgeons and an anorectal malformation is very difficult to simulate in an animal and not possible for in office or at home training.

It is general knowledge that artificial training models are not as realistic as the performing the procedure in the clinical setting on a patient, because of the lack of true haptic sensations. This is, therefore, not the focus of the training model. The focus is on learning and obtaining the steps of the total procedure and to adopt a structured approach to the correction of an anorectal malformation with the sagittal procedure.

Other inanimate models have been evaluated in similar questionnaire studies to establish the face (realism of the simulated tasks) and content (didactic value) validity. For this ARM model both the face and content validity are proven in this study. Because there was no other inanimate PSARP previously validated, or described to be developed, the outcome of this study could not be compared with other similar models. Therefore, the results of this validity study on our PSARP model was compared with the results of validation studies of other inanimate models used in (pediatric) surgical training. Including laparoscopic simulators, which have been the focus of training and development in general surgery for decades already. These results show that the use of real instruments and sutures enhances the realism and training value of the simulators (realism real laparoscopic suturing means 3.9–4.4 versus virtual reality 1.8–2.5, *p* < 0.001), even if not all specifics of the trained procedure are similar to the clinical setting [13–17]. The laparoscopic fundoplication model, developed by Botden et al., even had means of 2.3–3.7 for realistic haptic feedback of the stomach and crura, but was still considered a valuable inanimate training option for surgical residents (mean 4.3) [7, 18]. Several minimally invasive esophageal atresia models were developed, including those by Barsness et al. which showed relatively low realism scores as well (means 3.7–3.8), but good training values and relevance of the training (means 4.2–4.7) [19–21]. The minimally invasive congenital diaphragmatic hernia model, also developed by Barsness et al., showed a mean realism 3.5–3.9 on a 5-point Likert scale and global opinion 2.8 on a 4-point Likert scale, but a 4.3 on the value of the model for training [15]. This showed that this ARM model scored high on many aspects, compared with the other mentioned training models [7, 13–21], in which the haptics and realism of our model are particularly good compared to the other models. Which indicates that this model is a very good training tool, which is likely to be used by trainees for preclinical practice or implementation in hands-on trainings.

### Limitations and future developments

During this study, we primarily focused on the PSARP procedure. However, several other procedures are known to repair the various forms of ARM, such as the Anterior Sagittal Anorectoplasty (ASARP) that spares the posterior muscle group. Although this study primarily focused on the PSARP-procedure, it is possible to practice the steps of the ASARP-procedure on this model as well. Moreover, the model represented only a small part of the ARM spectrum, with the disposable sponge representing the perineal body with a rectoperineal fistula in a female patient. Consequently, this meant that not every step/variant of the PSARP procedure could be trained using this model, such as dissection and ligation of a recto-urethral fistula. Therefore, future developments will focus will be on a recto-vestibular fistula and the creation of a male model with a urethral fistula. With these adaptations several variants of the PSARP/ ASARP procedure can be trained on the model. In addition, an instruction video will enhance the training possibility in the home-based setting. Although the model was regarded a very good tool for training, the dissection of the fistula was regarded as less realistic. Because the focus was on the training of the component steps in an affordable model and not a perfect dissection model, this was an expected outcome. Future developments will aim to improve the dissection of the fistula, however, we do feel it is important to make sure it will still be affordable.

However, this model is an artificial model and as it is with animal models, they will only reflect the clinical situation. Although an artificial model is not able to replace the training of the procedure in the clinical setting, it will be an additional tool within the learning curve of the PSARP/ ASARP procedure. An important aspect in the learning curve of the clinical setting is the decision making, which is specifically needed during the dissection of the fistula and sometimes even finding the fistula. This cannot be trained on this model, although the use of E-learnings or video training could be beneficial in training these decisions as an additive to the model.

## Conclusion

We developed an affordable and partially reusable simulation model for the training of the basic component steps of the repair of an anorectal malformation with perineal fistula. This model is considered a good training tool and new asset in the training of pediatric surgeons and can be used to train the posterior/ anterior sagittal anorectoplasty outside the clinical setting.

## Data Availability

The datasets used and/or analysed during the current study are available from the corresponding author on reasonable request.

## Abbreviations

ARM: Anorectal malformation
PSARP: Posterior Sagittal Anorectoplasty
ASARP: Anterior Sagittal Anorectoplasty
SPSS: Statistical Package for Social Sciences
